# Author’s Response to Peer Reviews of “A Full-Scale Agent-Based Model to Hypothetically Explore the Impact of Lockdown, Social Distancing, and Vaccination During the COVID-19 Pandemic in Lombardy, Italy: Model Development”

**DOI:** 10.2196/32798

**Published:** 2021-09-10

**Authors:** Giuseppe Giacopelli

**Affiliations:** 1 Department of Mathematics and Informatics University of Palermo Palermo Italy

**Keywords:** epidemiology, computational, model, COVID-19, modeling, outbreak, virus, infectious disease, simulation, impact, vaccine, agent-based model

*This is the author’s response to peer-review reports for “A Full-Scale Agent-Based Model to Hypothetically Explore the Impact of Lockdown, Social Distancing, and Vaccination During the COVID-19 Pandemic in Lombardy, Italy: Model Development”.*

## Round 1 Review

The authors of the manuscript [1] are grateful to the editor and reviewers [2,3] for their invaluable input and feedback.

### Anonymous [2]

#### Major Comments

1. I agree with the reviewer, but the underlying idea of the paper is to create a model using just simple open-access data, like population density and estimations made on publicly available ensemble data (eg, number of contagions, number of deaths, etc). The author knows perfectly that this amount of data is not sufficient to fully depict epidemic behavior, but the idea is that on a very large scale, this information along with a fitting on the free parameters can approximate epidemic behavior. This has been explained better:

“The random walk behavior must be intended as an approximation of the actual motion of people during the day; this approximation was introduced to reduce the amount of information required to run the model and is widely used in many fields of science (eg, ideal gas theory)…”

2. References to previous models have been added:

“In particular, agent-based modeling in epidemiology has been used widely in the past. However, due to its computational limitations, approaches based on differential equations like SIR (susceptible-infected-recovered) models have often been preferred. The latest advances in computer science and engineering and the outbreak of COVID-19 have led to the use of ABM for simulating small community epidemic behavior…”

3. The model has been compared with an SIRD (susceptible-infected-recovered-deceased) model fitted on the outbreak scenario in terms of the rooted mean squared error in the infected, recovered, and deaths curves, outperforming the SIRD model in fitting the recovered curve and obtaining higher but comparable distances in the infected and deaths curves. It must be pointed out that even if the performance of the proposed model is comparable to the SIRD model, it has many advantages over the SIRD model (as pointed out in the paper):

“The proposed model has been compared with the classical SIRD model [33] fitted with parameter exploration on outbreak data. The result can be seen in Figure 2. It can be seen that the results are comparable: in terms of the rooted mean squared error from the data, the SIRD model has an error of 150 for the infected, 71 for the recovered, and 18 for deaths. The proposed model exhibits an error of 535 for the infected, 58 for the recovered, and 34 for deaths. This means that the model has comparable performance with the SIRD model (outperforming in the recovered), but it is not ODE mediated, so it is suitable to test alternative scenarios.”

4. This is true, but it can be approximated (like in ideal gas theory) with the idea that even if the behavior of each person is not random, the interaction for large numbers of people can be approximated with a random walk. Future work could aim to reconstruct people’s behavior in a more realistic way; however, it would require additional data (usually covered by privacy laws) that are not in the public domain. This would, unfortunately, contradict the aim of the paper of constructing a model based on public-domain information, making the model available for anyone. So, the random walk should be intended as an assumption and not as a ground truth.

“The random walk behavior must be intended as an approximation of the actual motion of people during the day; this approximation was introduced to reduce the amount of information required to run the model and is widely used in many fields of science (eg, ideal gas theory)…”

5. The following has been added to the paper:

“The creation of this algorithm was a challenging aspect of this study. The idea was to use matrix optimization in order to speed up the computation. The territory was subdivided into 20-km–long cells, and the cells in every frame were completely independent, with the supposition that, on average, every cell contains *m* people. In order to compute the distance between all nodes in the network, we had to compute the order of *N*^2^ pairwise distances.

“With this scheme, we had to only compute the order of *m*^2^ distances for each block multiplied by the number of blocks (which is about *N/m*) that is an order of *Nm*. Considering *m* small in comparison with *N*, it can be said that the scheme has a complexity near the order of *N* (for large *N* and small *m*). However, determining in which cell a person is located was also challenging because of the large size of the population. For these reasons, a simple grid scheme was used to locate nodes inside the cells. We used the following idea—supposing a segment from 0 km to *L_C_=*2 km with *N_c_=*4 cells:

1. From 0 km to 0.5 km
2. From 0.5 km to 1 km
3. From 1 km to 1.5 km
4. From 1.5 km to 2 km

“If, for example, the point *p*=0.6 km needed to be located, the formula used to calculate this would be *id_p_*=*ceil*(*N_c_p*/*L_c_*). The result is 2, indicating the second cell. Applying this formula for the x-axis and y-axis allows the algorithm to locate people in the cells. Although this algorithm may appear to be simple, it requires few calculations to be computed, which can make a substantial difference when a large number of agents is concerned.”

6. The density of the population has been taken into account from publicly available data. Additionally, the variation in the density of the population has been taken into account, but because of the limited length of the daily path of the nodes in their random walk, the variability in population density is not very notable. This work has not accounted for the demographic profile of the population, but because of the agent-based nature of the model, it could be easily implemented (given an accurate spatial demographic profile and detailed demographic-dependent statistics about COVID-19, which are not publicly available to the best of the author’s knowledge). This has been pointed out in the following:

“The displacement of the particles follows the density of inhabitants in Lombardy (ie, publicly available data). Even if more accurate data on people displacement and movement could be used, privacy concerns may not permit the open-source and open-access distribution of this data.”

#### Minor Comments

1. The paper has been revised.

2. The abstract has been revised.

3. Fixed in the text:

“Figure 1: The 3-layer structure of the model. The first layer, environment and agents, represents the motion of the inhabitants. The second layer represents social interaction between people in terms of collision detection. The third layer represents the virus dynamic in terms of epidemic behavior.”

4. The paper has been organized into Introduction, Methods, Results, and Discussion.

### Anonymous [3]

#### Major Comments

1. The language of the paper has been revised.

2. The model has been depicted more clearly in terms of a mathematical description. The model, as it is, outlines a general procedure to approach an epidemic. Most of the data used to create the model are realistic, starting from geographic distribution (that is, as realistic as possible to reproduce population displacement but, on the other hand, is not demanding in terms of the data required to run the model) and virus characterization. In the text, I have underlined a general procedure to deploy the model in different scenarios:

“Moreover, in this paper, since most of the parameters are realistic, the model can be run for a general epidemic upon collecting the few parameters required (which in this case were all open access) and fitting the two parameters left. However, the model can be made more precise by adding additional realistic data, which most of the time are not fully open access; this, however, is out of the scope of this study.”

3. I thank the reviewer for this comment because I think it is the key point of the paper. More details have been added (see response #5 to Anonymous [2]).

#### Minor Comments

1. I agree with the reviewer, and I have added the following to the Future Works section:

“This work provides a novel, efficient, and low-demanding (in terms of computational resources) population model. Many features remain to be introduced in the model, like an age-dependent virus model, the ability to introduce an age parameter in the model or a more precise spatial simulation based on big data, and the ability to simulate the habits of the population. In conclusion, future work could be done to increase the number of frames per day, thereby improving the performance of the agents.”
